# Complete Genome Sequences of Two Methicillin-Sensitive *Staphylococcus aureus* Isolates Representing a Population Subset Highly Prevalent in Human Colonization

**DOI:** 10.1128/genomeA.00716-16

**Published:** 2016-07-28

**Authors:** Robert E. Weber, Franziska Layer, Stephan Fuchs, Jennifer K. Bender, Stefan Fiedler, Guido Werner, Birgit Strommenger

**Affiliations:** National Reference Centre for Staphylococci and Enterococci, Division Nosocomial Pathogens and Antibiotic Resistances, Department of Infectious Diseases, Robert Koch Institute, Wernigerode Branch, Wernigerode, Germany

## Abstract

Here, we report the high-quality draft genome sequences of two methicillin-susceptible *Staphylococcus aureus* isolates, 08-02119 and 08-02300. Belonging to sequence type 582 (ST582) and ST7, both isolates are representatives of clonal lineages often associated with asymptomatic colonization of humans.

## GENOME ANNOUNCEMENT

Methicillin-susceptible *Staphylococcus aureus* (MSSA) strains are common human colonizers. Up to 80% of humans carry *S. aureus*, with approximately 20% being persistent carriers. Asymptomatic carriage increases the risk of endogenous infection, especially in the hospital environment (1).

The MSSA population is highly diverse in comparison to the population of hospital-associated methicillin-resistant *S. aureus* (HA-MRSA). However, predominating lineages include sequence type 7 (ST7) and clonal complex 15 (CC15) (2, 3). While ST7 is rarely associated with disease, CC15 isolates frequently carry the phage-encoded exfoliation gene *eta*, which causes the presentation of exfoliative dermatitis (4). Isolates 08-02119 and 08-02300 belong to CC15 (ST582, single-locus variant [SLV] of ST15) and ST7, respectively; both were cultured from wound infections in 2008. Their genome sequences will contribute to studies on the comparative genomics of prevalent MSSA lineages and host interaction in human carriage.

Whole-genome sequences were generated by GATC Biotech (Konstanz, Germany) using a PacBio RS II sequencer (Pacific Biosciences, USA). *De novo* assembly utilizing HGAP3 (Pacific Biosciences) yielded one contig each, with 282- and 322-fold coverage. To confirm PacBio results, Illumina sequencing was performed on a MiSeq using the 2 × 300-cycle version 3 kit, as recommended by the manufacturer (Illumina, San Diego, CA, USA). Illumina reads were mapped to the PacBio contigs by the use of Geneious version 8.1.7 (Biomatters Ltd., Auckland, New Zealand). Ring closures were verified by PCR. The genome sequences of *S. aureus* 08-02119 and 08-02300 are 2,796,894 bp and 2,742,807 bp in length, respectively. According to the NCBI Prokaryotic Genome Annotation Pipeline (5) 2,829 genes and 2,747 coding sequences were predicted for *S. aureus* 08-02119, while *S. aureus* 08-02300 contains 2,756 genes and 2,674 coding sequences. Both isolates exhibit 19 rRNAs, 59 tRNAs, and a G+C content of 32.9%. The genome sequences were subjected to the VirulenceFinder and ResFinder tools provided by the Center for Genomic Epidemiology (CGE) (6, 7). In 08-02119, the penicillin resistance gene *blaZ* was detected and confirmed by PCR. *S. aureus* 08-02300 does not harbor any resistance genes. The genotypic results were concordant with the results of susceptibility testing.

VirulenceFinder detected *aur, spIA, spIB, spIE, scn, lukD, lukE, hlb, hlgA, hlgB*, and *hlgC* in both genomes. Further, the exfoliative toxin A gene (*eta*) was located within an intact prophage (NC_028859) in isolate 08-02119. Isolate 08-02300 harbors *sak, sea,* and *sep*. *sea*, s*cn*, and *sak* are prophage encoded (NC_008617).

PacBio sequencing did not indicate the presence of plasmids, which was verified by resolving S1 nuclease-treated genomic DNA via pulsed-field gel electrophoresis (data not shown). The PHAge Search Tool (PHAST [8]) was applied for the identification and annotation of prophage sequences. In isolate 08‑02119, two of three prophage regions were intact (positions 578749 to 657660 [NC_009762] and 1314536 to 1360560 [NC_028859]; incomplete, positions 1929685 to 1938122 [NC_021323]); in isolate 08-02300, four prophage regions were identified, of which one was intact (positions 2108983 to 2157602 [NC_008617]; incomplete, positions 407256 to 415795 [NC_021323], 1409855 to 1419493 [NC_019914], and 1957745 to 1788804 [NC_023500]).

### Nucleotide sequence accession numbers.

The genome sequences of *S. aureus* 08-02119 and 08-02300 have been deposited in GenBank under the accession numbers CP015645 and CP015646, respectively.
